# Protein kinase D1 regulates ERα-positive breast cancer cell growth response to 17β-estradiol and contributes to poor prognosis in patients

**DOI:** 10.1111/jcmm.12322

**Published:** 2014-10-07

**Authors:** Manale Karam, Ivan Bièche, Christine Legay, Sophie Vacher, Christian Auclair, Jean-Marc Ricort

**Affiliations:** a Laboratoire de Biologie et de Pharmacologie Appliquée, UMR 8113 CNRS, Ecole Normale Supérieure de CachanCachan, France; b Laboratoire d'Oncogénétique, Institut Curie – Hôpital René Huguenin, FNCLCCSaint-Cloud, France

**Keywords:** protein kinase D1, estrogen receptor α, breast cancer, proliferation, invasion, antiestrogen therapy

## Abstract

About 70% of human breast cancers express and are dependent for growth on estrogen receptor α (ERα), and therefore are sensitive to antiestrogen therapies. However, progression to an advanced, more aggressive phenotype is associated with acquisition of resistance to antiestrogens and/or invasive potential. In this study, we highlight the role of the serine/threonine-protein kinase D1 (PKD1) in ERα-positive breast cancers. Growth of ERα-positive MCF-7 and MDA-MB-415 human breast cancer cells was assayed in adherent or anchorage-independent conditions in cells overexpressing or depleted for PKD1. PKD1 induces cell growth through both an ERα-dependent manner, by increasing ERα expression and cell sensitivity to 17β-estradiol, and an ERα-independent manner, by reducing cell dependence to estrogens and conferring partial resistance to antiestrogen ICI 182,780. PKD1 knockdown in MDA-MB-415 cells strongly reduced estrogen-dependent and independent invasion. Quantification of PKD1 mRNA levels in 38 cancerous and non-cancerous breast cell lines and in 152 ERα-positive breast tumours from patients treated with adjuvant tamoxifen showed an association between PKD1 and ERα expression in 76.3% (29/38) of the breast cell lines tested and a strong correlation between PKD1 expression and invasiveness (*P* < 0.0001). In tamoxifen-treated patients, tumours with high PKD1 mRNA levels (*n* = 77, 50.66%) were significantly associated with less metastasis-free survival than tumours with low PKD1 mRNA expression (*n* = 75, 49.34%; *P* = 0.031). Moreover, PKD1 mRNA levels are strongly positively associated with EGFR and vimentin levels (*P* < 0.0000001). Thus, our study defines PKD1 as a novel attractive prognostic factor and a potential therapeutic target in breast cancer.

## Introduction

Protein kinase D1 (PKD1), encoded by the *PRKD1* gene and formerly called protein kinase Cμ (PKCμ), is a serine/threonine kinase which is implicated in the regulation of a complex array of fundamental biological processes, including signal transduction, membrane trafficking, cell proliferation, survival and differentiation, migration, angiogenesis and cancer [1–3]. Signalling through PKD1 is induced by a remarkable number of stimuli, including G-protein-coupled receptor agonists and growth factors. Through PLC-mediated hydrolysis of phosphatidylinositol 4,5-biphosphate, they activate PKD1, which appears both as a direct target of diacylglycerol (DAG) and as a downstream target of protein kinase C isoforms [4,5]. Active PKD1 regulates cancer related signalling pathways such as mitogen-activated ERK kinase/extracellular signal-regulated kinase (MEK/ERK), nuclear factor-kappa B (NFκB) and histone deacetylase (HDAC) pathways [3,6]. PKD1 has a complex relationship with respect to cancer development. In fact, depending on the tissue type, different PKD1 expression alterations and consequences were observed [3]. To date, in breast cancer, the role of PKD1 remains unclear.

In the mammary gland, estrogens are potent mitogens that play a pivotal role in the initiation and progression of carcinoma [7]. They mostly act through their nuclear receptor (i.e. estrogen receptor α; ERα) the activation of which can lead to breast carcinogenesis by stimulating tissue growth and inhibiting apoptosis. About 70% of human breast cancers express ERα. Therefore, they require estrogens for proliferation and survival, and are sensitive to antiestrogen therapies such as tamoxifen [8–10]. However, in advanced disease cases, many ERα-positive tumours progress into an estrogen-independent and antiestrogen-resistant phenotype, a hallmark of breast cancer with poor prognosis, often resulting in tumour progression and mortality [11]. ERα increases proliferation and survival by functioning as ligand-activated transcription factor or as signal transductor [12,13]. Molecular partners downstream of growth factor receptors, such as type I insulin-like growth factor receptor (IGF-IR), epidermal growth factor receptor (EGFR) and some G-protein-coupled receptors (GPCR), can also activate ERα in a ligand-independent manner. Moreover, ERα activity can be modulated by post-translational modifications such as its phosphorylation onto multiple residues [14]. Therefore, ERα phosphorylation induced by 17β-estradiol onto Ser118, and to a lesser extent onto Ser104 and Ser106, or onto Ser118 and Ser167 following the activation of multiple kinases such as ERK1/2 enhances its function [15–18].

PKD1 promotes major phenotypic changes in ERα-positive MCF-7 cells [6]. Among others, PKD1 overexpressing cells acquire the ability to grow independently of anchorage and to form tumours in nude mice. Since MCF-7 cells are estrogen-dependent and non-tumorigenic unless exogenous estrogen is provided to the mice [19], we determined in the present study whether PKD1 regulates cell sensitivity and/or dependence to estrogens in two different ERα-positive breast cancer cell lines. Furthermore, to confirm and understand the role of PKD1 in breast cancer, we analysed the expression pattern of PKD1 mRNA in a series of 38 non-cancerous or malignant breast cell lines and 152 ERα-positive breast tumours from tamoxifen-treated patients with long-term follow-up and its association with tamoxifen responsiveness and classical clinicopathological prognostic factors.

## Materials and methods

### Antibodies and materials

The following antibodies were used at the indicated dilutions: anti-PKD1 (sc-935; 1/500) and anti-α-actinin (1/5000) were from Santa Cruz Biotechnology (Santa Cruz, CA, USA), anti-phospho-PKD1 (1/1000), anti-cleaved PARP (1/1000), anti-ERα (1/2000), anti-phospho-S118-ERα (1/2000) and anti-phospho-S167-ERα (1/2000) were from Cell Signaling Technology (Danvers, MA, USA). Horseradish peroxidase-conjugated secondary antibodies used were goat anti-rabbit IgG (1/2000; Dako, Glostrup, Denmark) and goat anti-mouse IgG (1/5000; Rockland, Gilbertsville, PA, USA). PKD1-targeting (sc-36245) and control non-targeting (sc-37007) siRNAs were purchased from Santa Cruz Biotechnology. 17β-estradiol, ICI 182,780, MTT and all other biochemicals were from Sigma-Aldrich (St. Louis, MO, USA).

### Patients and samples

Samples of primary breast tumours excised from 152 women at Centre René Huguenin (Saint-Cloud, France) from 1978 to 2008 and immediately stored in liquid nitrogen until RNA extraction were analysed. All patients who entered our institution before 2007 were informed that their tumour samples might be used for scientific purposes and had the opportunity to decline. Since 2007, patients entering our institution have given their approval also by signed informed consent. This study was approved by the local ethics committee (Breast Group of René Huguenin Hospital).

All patients (mean age 69 years, range 52–86) met the following criteria: primary unilateral non-metastatic breast carcinoma; ERα positivity; complete clinical, histological and biological data available; no chemotherapy before surgery; and full follow-up at Centre René Huguenin. Treatment consisted of modified radical mastectomy in 114 cases (75%) or breast-conserving surgery plus locoregional radiotherapy in 38 cases (25%), then all patients received post-operative adjuvant endocrine therapy (tamoxifen, 20 mg daily for 3–5 years), and no other treatment. Patients underwent physical examination and routine chest radiography every 3 months for 2 years, then annually. Mammograms were done annually.

Estrogen receptor α, progesterone receptor (PR) and human epidermal growth factor receptor 2 (ERBB2) statuses were determined at the protein level by using biochemical methods (dextran-coated charcoal method, enzyme immunoassay or immunohistochemistry) and confirmed by real-time quantitative PCR assays [20,21]. During a median follow-up of 10 years (range 13 months to 23 years), 57 patients developed metastasis. Ten specimens of adjacent normal breast tissue from breast cancer patients (*n* = 2) and normal breast tissue from women undergoing cosmetic breast surgery (*n* = 8) were used as sources of normal RNA [22].

### Cell culture

Breast tissue derived cell lines were obtained from the American Type Culture Collection (ATCC, Manassas, VA, USA) or from the German Resource Centre for Biological Material (DSMZ, Braunschweig, Germany). For PKD1 expression and clinicopathological analyses, each of the 38 cell lines tested was cultured in the conditions recommended by ATCC. For the other *in vitro* experiments, MCF-7 and MDA-MB-415 cell lines were cultured as follows. The MCF-7 cell line was cultured in DMEM-Glutamax®, supplemented with 10% foetal bovine serum (FBS) and 100 units/ml penicillin and 100 μg/ml streptomycin (P/S); complete medium (Invitrogen-Life Technologies, Cergy-Pontoise, France). 1 mg/ml G418 (Calbiochem, Darmstadt, Germany) was added to this medium for stably transfected MCF-7 cell culture. The MDA-MB-415 cell line was cultured in complete medium supplemented with 10 μg/ml insulin and 10 μg/ml glutathione. Prior to experiments, cells were cultured for 48 hrs in estrogen-free medium that corresponds to the complete medium in which phenol red-free DMEM and charcoal-treated FBS were used.

### Stable transfection

80–90% confluent MCF-7 cells cultured in Opti-MEM I medium were transfected with 12 μg of either pcDNA3 (Invitrogen-Life Technologies) or pcDNA3-PKD1 (generous gift from Dr F.J. Johannes, University of Stuttgart, Germany) plasmids and 60 μg of Lipofectamin™ 2000 reagent according to manufacturer's protocol and as previously described [6]. A pool of two clones stably overexpressing PKD1 (P1 and P2 cells) and a pool of two clones transfected with the empty pcDNA3 vector (C1 and C2 cells) were used.

### siRNA transfection

siRNA transfection was done according to manufacturer's protocol and as previously described [6]. After transfection, estrogen-free medium was added and cells cultured for an additional 18–24 hrs at 37°C before analysis.

### Western Blot analysis

Total proteins were extracted and quantified as previously described [6]. 30–80 μg of total protein extracts were separated by SDS-PAGE and transferred onto nitrocellulose membranes. These were incubated with the specific antibodies overnight at 4°C and revealed by enhanced chemiluminescence (Amersham, GE Healthcare, UK).

### MTT assay

Cell survival and proliferation were determined by the MTT assay. Transfected MCF-7 and MDA-MB-415 cells were seeded in quadruplicates into 96-well plates at a density of 4–6 × 10^3^ cells per well in estrogen-free medium and allowed to adhere overnight at 37°C. Then, cells were incubated for different periods of time with the same medium containing or not different concentrations of 17β-estradiol, ICI 182,780 or vehicle (ethanol or DMSO) before MTT assay as previously described [6].

### 5-bromo-2′-deoxyuridine (BrdU) incorporation assay

Cells were seeded in quadruplicates into 96-well plates at the density of 5000 cells per well in estrogen-free medium containing 10% charcoal-treated FBS and incubated for 35 hrs. Cells were then incubated with the same medium containing or not different concentrations of 17β-estradiol for 24 hrs before addition of BrdU for 4 hrs. Proliferation was then assessed by examining BrdU incorporation by using the BrdU Cell Proliferation Assay Kit (Cell Signaling Technology) according to manufacturer's instructions.

### Colony formation assay

MCF-7 and MDA-MB-415 cells (10,000 cells) were resuspended in 2.5 ml of methylcellulose (0.8%) prepared in estrogen-free medium containing or not different concentrations of 17β-estradiol or vehicle (ethanol). Cells plated in uncoated 35 mm culture dishes were incubated for 3 or 6 weeks. Plates were then photographed and macroscopic colonies counted.

### Transwell invasion assay

24-well transwell chambers (Corning Costar, Corning, NY, USA) with 8.0-μm pore size polycarbonate membrane were used. 100,000 cells were plated in duplicates in 200 μl of phenol red-free DMEM medium in the upper well which was coated with 50 μl phenol red-free matrigel (BD Biosciences, San Jose, CA, USA) diluted to 1 mg/ml. Complete medium was added to the lower well. After 48 hrs incubation, cells that migrated through the membrane to the lower well were fixed, stained with 0.05% crystal violet and then all counted by light microscopy.

### RNA extraction

Total RNA was extracted from breast cell lines and tumour samples by using acid-phenol guanidium as previously described [23]. RNA quality was determined by electrophoresis through agarose gels, staining with ethidium bromide and visualization of the 18S and 28S RNA bands under ultraviolet light.

### Real-time RT-PCR

qRT-PCR was performed with the ABI Prism 7900 sequence detection system (Perkin-Elmer Applied Biosystems, Foster City, CA, USA) and PE biosystems analysis software according to the manufacturer's manuals. RNA levels were normalized to the level of TBP (TATA box-binding protein) and calculated as N_*PRKD1*_ = 2^ΔCtsample^. The detailed procedures of qRT-PCR were followed as previously described [24]. The primers for *TBP* and *PRKD1*, *ESR1*, *PR* and *ERBB2* genes were chosen with the assistance of the Oligo 6.0 program (National Biosciences, Plymouth, MN, USA). dbEST and nr databases were scanned to confirm the total gene specificity of the nucleotide sequences chosen for the primers and the absence of single nucleotide polymorphisms. Primer sequences for *PRKD1* are ACAGGAACCAACTTGCACAGAGAT (PRKD1-U2) and GTGCTGATGTCCACATTTTCTTGA (PRKD1-L2), those of the other genes are as previously indicated [24]. To avoid amplification of contaminating genomic DNA, one of the two primers was placed at the junction between two exons. Agarose gel electrophoresis was used to verify the specificity of PCR amplicons.

### Statistical analysis

For *in vitro* experiments, statistical significance of differences between experimental groups was performed by Student's *t*-test by using the GraphPad Prism 5.03 software (San Diego, CA, USA). *P*-values <0.05 (*) were considered significant. The distributions of PKD1 mRNA levels in breast samples were characterized by their median values and ranges. Relationships between mRNA levels of the different target genes, and between mRNA levels and clinical parameters, were identified by using non-parametric tests, namely chi-square test (relation between two qualitative parameters), Mann–Whitney *U*-test (relation between one qualitative parameter and one quantitative parameter) and Spearman rank correlation test (relation between two quantitative parameters). Differences were considered significant at confidence levels greater than 95% (*P* < 0.05).

To visualize the efficacy of a molecular marker (PKD1 mRNA level) to discriminate between two populations (patients that developed/did not develop metastases) in the absence of an arbitrary cut-off value, data were summarized in a ROC (receiver operating characteristic) curve [25]. The area under curve was calculated as a single measure to discriminate efficacy. Metastasis-free survival (MFS) was determined as the interval between initial diagnosis and detection of the first metastasis. Survival distributions were estimated by the Kaplan–Meier method, and the significance of differences between survival rates were ascertained with the log-rank test.

## Results

### PKD1 induces estrogen-dependent MCF-7 breast cancer cell proliferation and survival in estrogen-free medium

**Fig. 1:**
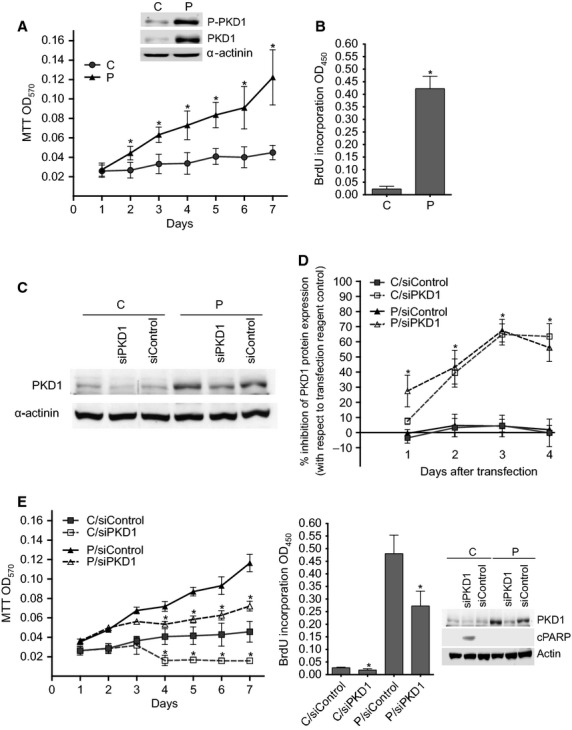
PKD1 induces MCF-7 cell proliferation and survival in estrogen-free medium. (**A**) PKD1-overexpressing (P) and control (C) cells were incubated for different periods of time (1–7 days) in estrogen-free medium. Viable cells were identified over seven days by MTT assay. The results presented are the means ± SD for four independent experiments. *P < 0.01 *versus* control cells. Inset: immunodetection of phospho-PKD1, PKD1 and α-actinin in cell lysates from C and P cells cultured in estrogen-free medium. (**B**) Control (C) and PKD1-overexpressing (P) MCF-7 cells were incubated for 48 hrs in estrogen-free medium before BrdU incorporation analysis. The results presented are the means ± SD for two independent experiments. *P < 0.01 *versus* control cells. (**C** and **D**) Control (C) and PKD1-overexpressing (P) MCF-7 cells were transfected with siPKD1 or siControl and cultured in phenol red-free DMEM containing 10% charcoal-treated FBS. From one to four days after transfection, proteins from transfected cells were subjected to SDS-PAGE, transferred to nitrocellulose and immunodetected with anti-PKD1 or anti-α-actinin antibodies. (**C**) The autoradiograms presented are those of typical experiments performed 4 days after transfection. (**D**) The percentage of inhibition of PKD1 expression was determined over 4 days after transfection. The results presented are the means ± SD for three independent experiments. *P < 0.01 *versus* transfection reagent treated cells. (**E**) PKD1-overexpressing (P) and control (C) cells were transfected with siPKD1 or siControl. The next day, cells were cultured in estrogen-free medium for different periods of time and then analysed as in panels A and B by MTT and BrdU incorporation assays. The results presented are the means ± SEM or SD for three or two independent experiments, respectively. *P < 0.01 *versus* transfection reagent treated cells. Inset: immunodetection of PKD1, cleaved PARP (cPARP) and actin in cell lysates from C and P cells transfected with siControl or siPKD1 and cultured for 3 days in estrogen-free medium.

To determine whether PKD1 might modulate the proliferation of estrogen-dependent MCF-7 cells in estrogen-free medium, growth of control (C) and PKD1-overexpressing (P) cells was analysed in phenol red-free DMEM supplemented with 10% charcoal-stripped FBS by MTT (Fig. 1A) and BrdU incorporation (Fig. 1B) assays. Results showed that, whereas control cells remained rather quiescent, PKD1-overexpressing cells still significantly proliferated in estrogen-free medium (significant induction of BrdU incorporation in P cells but not in C cells, Fig. 1B) and showed ∼4.5-fold increase in cell number after 7 days of culture (Fig. 1A).

To determine whether this proliferation was specifically because of PKD1 overexpression, PKD1 expression was inhibited by transfecting the cells with either specific PKD1-targeting (siPKD1) or control non-targeting (siControl) siRNAs. As shown in Figure 1C and D, whereas siControl had no significant effect, siPKD1 significantly decreased PKD1 protein expression in both cell lines with a maximal effect (about 68%) obtained 3–4 days after transfection. Noteworthy, proliferation in estrogen-free medium of PKD1-overexpressing cells was not affected by control siRNAs but significantly reduced when transfected with PKD1-targeting siRNAs (Fig. 1E) clearly demonstrating that PKD1 overexpression allows estrogen-dependent MCF-7 breast cancer cells to proliferate in estrogen-free medium. Interestingly, siPKD1, but not siControl, seems to induce the death of control cells as shown by associated induction of PARP cleavage (Fig. 1E), indicating that endogenous PKD1 is also necessary for MCF-7 cell survival in estrogen-free medium.

### PKD1 increases MCF-7 cell sensitivity to 17β-estradiol for proliferation

**Fig. 2:**
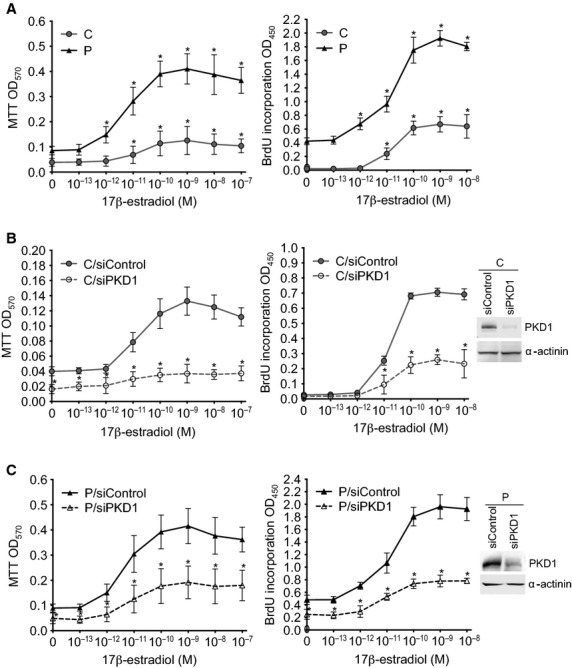
PKD1 overexpression increases MCF-7 cell sensitivity to estradiol for proliferation. PKD1-overexpressing (P) and control (C) MCF-7 cells transfected (**B** and **C**) or not (**A**) with siPKD1 (empty symbols) or siControl (plain symbols) were incubated in phenol red-free medium containing 10% charcoal-treated FBS and different concentrations of 17β-estradiol (10^−13^–10^−7^ M) for 4 days or 24 hrs before being analysed by MTT or BrdU incorporation assays, respectively. The results presented are the means ± SD for four (MTT assay) or two (BrdU incorporation assay) independent experiments. *P < 0.01 *versus* unstimulated cells (**A**) or *versus* transfection reagent treated cells (**B** and **C**). Insets: immunodetection of PKD1 and α-actinin in C (**B**) and P (**C**) cells after 3 days of transfection with siControl or siPKD1.

To determine whether PKD1 overexpression may increase MCF-7 cell sensitivity to estrogens for proliferation, MCF-7 cells overexpressing (P) or not (C) PKD1 were analysed by MTT or BrdU incorporation assays after being cultured in phenol red-free medium supplemented with 10% charcoal-stripped FBS and different concentrations of 17β-estradiol for 4 days or 24 hrs, respectively. 17β-estradiol dose-dependently increased proliferation of both control and PKD1-overexpressing cells with a maximal effect observed at 10^−10^–10^−9^ M (Fig. 2A). However, compared to control cells, PKD1-overexpressing cells started to proliferate at lower 17β-estradiol concentrations (10^−12^ *versus* 10^−11^ M), were more responsive (fivefold increase in cell number at 10^−9^ M 17β-estradiol *versus* threefold for untreated cells) and possessed higher proliferative rates whatever the 17β-estradiol concentration used is. Furthermore, siPKD1, but not siControl, strongly inhibited 17β-estradiol-induced proliferation of both control cells (72% and 48.57% decrease in cell number and BrdU incorporation, respectively; Fig. 2B) and PKD1-overexpressing cells (by 56% and 60% decrease in cell number and BrdU incorporation, respectively; Fig. 2C). Therefore, taken together, these results demonstrated that PKD1 overexpression increases MCF-7 cell sensitivity to 17β-estradiol for proliferation and that 17β-estradiol-induced MCF-7 cell proliferation is mediated, at least in part, by PKD1.

### PKD1 stimulates ERα expression in MCF-7 cells

**Fig. 3:**
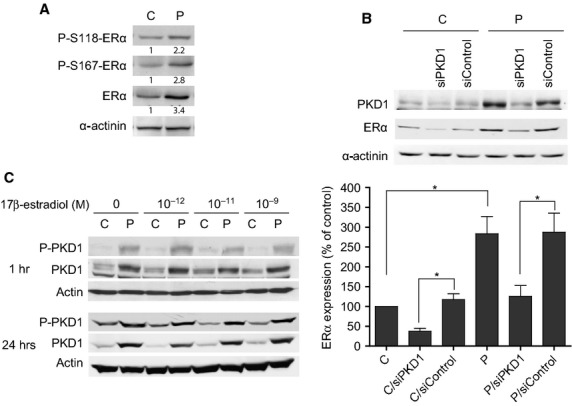
PKD1 enhances ERα expression in MCF-7 cells. (**A**) Control (C) and PKD1-overexpressing (P) MCF-7 cells cultured in estrogen-free medium were lysed and proteins analysed by western blot by using anti-phospho-ERα(S118) (P-S118-ERα), anti-phospho-ERα(S167) (P-S167-ERα), anti-ERα or anti-α-actinin antibodies. Values presented under each autoradiogram represent quantitative analysis of each band in fold increase compared to C cells. The results presented are those of typical experiments. (**B**) Control (C) and PKD1-overexpressing (P) MCF-7 cells transfected or not with siPKD1 or siControl were cultured for 3 days in estrogen-free medium and then analysed by western blot for PKD1, ERα and α-actinin expression. The autoradiograms presented are those of a typical experiment. The histograms represent quantitative analysis of ERα expression expressed as percentage of ERα expression measured in untreated control cells (C). The results presented are the means ± SEM for five independent experiments. *P < 0.01. (**C**) PKD1-overexpressing (P) and control (C) MCF-7 cells were stimulated for 1 or 24 hrs with different concentrations of 17β-estradiol. Cells were then lysed and proteins analysed by western blot by using anti-PKD1, anti-phospho-PKD1 or anti-actin antibodies. The results presented are those of a typical experiment.

To determine whether this increased proliferation of PKD1-overexpressing cells is associated with a regulation of the activity and/or expression of ER, ERα expression and phosphorylation state of its Ser118 and Ser167 residues were analysed. Compared to control cells, ERα expression was significantly increased in PKD1-overexpressing cells cultured in estrogen-free medium and as a consequence so was the level of phosphorylated ERα (Fig. 3A). Moreover, siPKD1, but not siControl, strongly inhibited ERα expression in both PKD1-overexpressing and control cells (∼56.3% and 68.1% inhibition, respectively; Fig. 3B). Therefore, our results demonstrated, for the first time, that PKD1 positively regulates ERα expression in MCF-7 cells.

Noteworthy, since ERα regulates gene transcription, we also investigated whether PKD1 expression could be modulated by 17β-estradiol. To address this issue, PKD1-overexpressing and control MCF-7 cells were stimulated with 17β-estradiol for 1 or 24 hrs before PKD1 expression analysis. We showed that PKD1 levels and phosphorylation state remained stable following 17β-estradiol treatment (Fig. 3C) demonstrating that 17β-estradiol does not affect PKD1 expression or activity.

### PKD1 confers partial resistance to the antiestrogen ICI 182,780

**Fig. 4:**
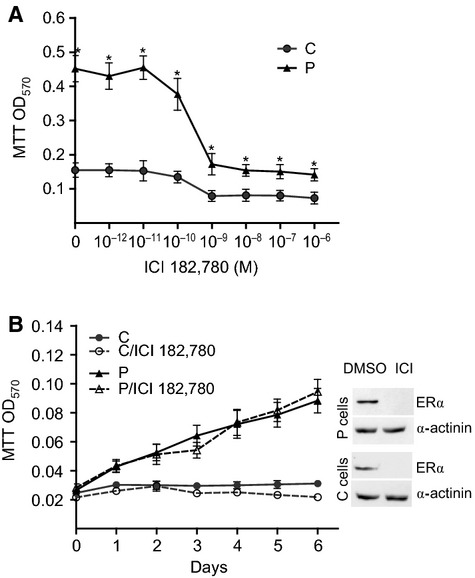
PKD1 overexpression confers a resistance to the antioestrogen ICI 182,780. (**A**) PKD1-overexpressing (P) and control (C) MCF-7 cells were incubated for 4 days in phenol red-free medium containing 10% charcoal-treated FBS, 10^−9^ M 17β-estradiol and different concentrations of ICI 182,780 (10^−12^–10^−6^ M), and then analysed by MTT assay. The results presented are the means ± SD for three independent experiments. *P < 0.01 *versus* control cells. (**B**) PKD1-overexpressing (P) and control (C) MCF-7 cells were incubated for different periods of time in estrogen-free medium containing 10^−7^ M ICI 182,780 or vehicle (DMSO) and then analysed by MTT assay. The results presented are the means ± SD for three independent experiments. Inset: immunodetection of ERα and α-actinin in cell lysates from P or C cells cultured for 24 hrs in estrogen-free medium containing 10^−7^ M ICI 182,780 (ICI) or DMSO.

To determine whether PKD1-increased cell proliferation is dependent upon the estrogen receptor, MCF-7 cells overexpressing or not PKD1 were incubated with the antiestrogen ICI 182,780 [26] and analysed for proliferation. As shown in Figure 4A, ICI 182,780 strongly inhibited 17β-estradiol-induced proliferation of control (total inhibition) and PKD1-overexpressing (70% inhibition) cells confirming the dependence of control MCF-7 cells to the estrogen receptor for proliferation and demonstrating that PKD1-enhanced sensitivity to 17β-estradiol is dependent upon the estrogen receptor.

Since PKD1-overexpressing cells still proliferate at high ICI 182,780 concentrations, we examined whether these cells had acquired a relative resistance to this antiestrogen. To answer this point, proliferation of control and PKD1-overexpressing cells was studied in estrogen-free medium in presence or not of 10^−7^ M ICI 182,780. As previously observed (Fig. 2A), control cells remained totally quiescent in estrogen-free medium and ICI 182,780 had, as expected, no supplementary effect (Fig. 4B). Noteworthy, although ICI 182,780 totally inhibited ERα expression in PKD1-overexpressing cells (Fig. 4B, inset), it did not affect their proliferation rate. This demonstrates that PKD1 overexpression allows MCF-7 cell proliferation and survival in an estrogen receptor-independent manner and therefore confers to the cells a relative resistance to the antiestrogen ICI 182,780.

### PKD1 enhances 17β-estradiol-induced and stimulates estrogen-independent anchorage-independent growth

**Fig. 5:**
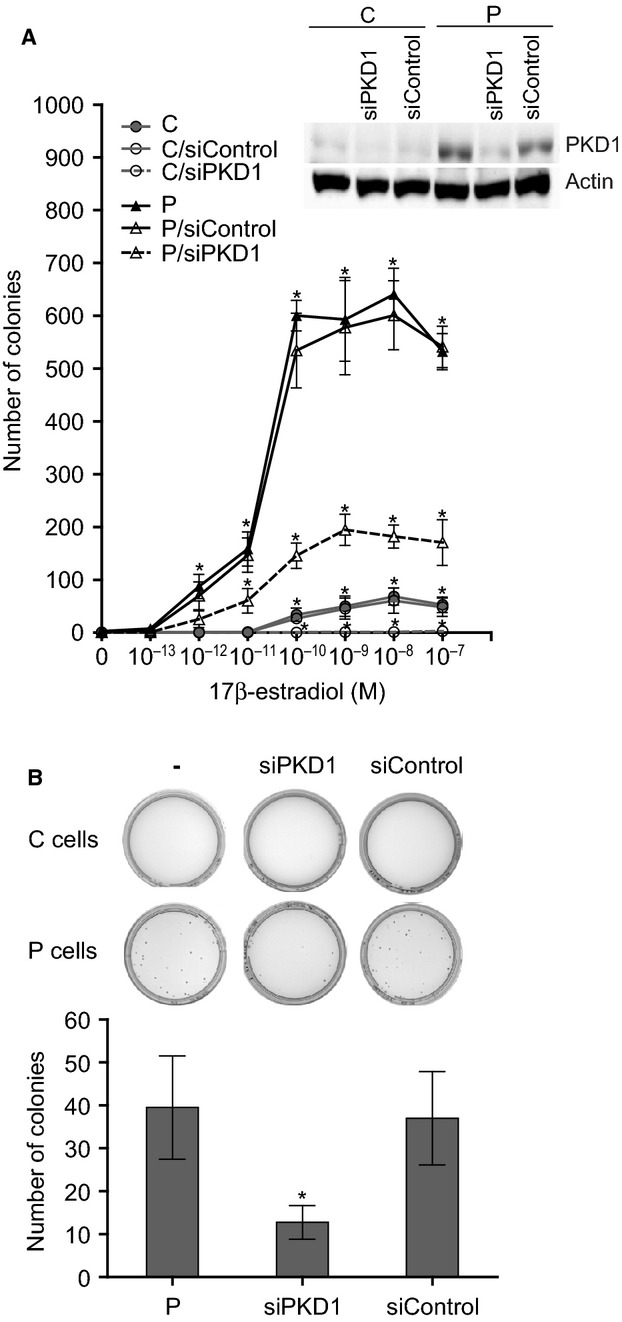
PKD1 overexpression increases sensitivity and reduces dependence of MCF-7 cells to 17β-estradiol for anchorage-independent growth. (**A**) PKD1-overexpressing (P) and control (C) MCF-7 cells transfected or not with siPKD1 or siControl were seeded in methylcellulose (10,000 cells per plate) containing different concentrations of 17β-estradiol (10^−13^–10^−7^ M) and colonies were counted after 3 weeks of culture. The quantifications presented are the means ± SD for four independent experiments. *P < 0.01 *versus* transfection reagent treated cells. Inset: immunodetection of PKD1 and actin in C and P cells after 3 days of transfection with siControl or siPKD1. (**B**) PKD1-overexpressing (P) and control (C) cells transfected or not with siPKD1 or siControl were cultured in estrogen-free methylcellulose and incubated for a longer period of time (5–6 weeks) before colony formation analysis. The photographs presented are those of a representative experiment. Histograms represent the quantitative analysis of P colonies and are the means ± SD for four independent experiments. *P < 0.01 *versus* transfection reagent treated cells.

PKD1 overexpression allows MCF-7 cells to proliferate in an anchorage-independent manner, which is a hallmark of oncogenic transformation, in estrogen-containing medium [6]. Therefore, taking into account our previous results (Figs 1 and 2), we examined whether this ability to proliferate independently of anchorage was dependent upon estrogens. As shown in Figure 5A, after 3 weeks of culture, neither control nor PKD1-overexpressing cells formed colonies in estrogen-free methylcellulose (0 M 17β-estradiol). By contrast, addition of 17β-estradiol to the culture medium dose-dependently promoted colony formation, which was detectable starting at 10^−10^ and 10^−12^ M 17β-estradiol for control and PKD1-overexpressing cells respectively. In addition to being more sensitive to 17β-estradiol than control cells, PKD1-overexpressing cells formed much more colonies whatever 17β-estradiol concentration used. Thus, at 10^−10^ M 17β-estradiol, a maximum of 600 colonies of PKD1-overexpressing cells was obtained which represents 10-fold the maximal number of colonies of control cells obtained at 10^−8^ M 17β-estradiol (Fig. 5A). Interestingly, siPKD1, but not siControl, strongly reduced colony formation of PKD1-overexpressing and control cells (by ∼68% and 98%, respectively; Fig. 5A). Thus, these results indicate that PKD1 plays a major role in 17β-estradiol-induced anchorage-independent growth.

Noteworthy, longer incubation times up to 5–6 weeks in estrogen-free medium allowed PKD1-overexpressing cells, but not control cells, to form colonies in methylcellulose (about 40 colonies over 10,000 seeded), which was largely impaired by siPKD1 but not by siControl (Fig. 5B). Taken together, these results suggest that PKD1 confers upon MCF-7 cells the ability to survive and grow in absence of anchorage not only by increasing their sensitivity to 17β-estradiol but also independently of this hormone.

### PKD1 mRNA expression in normal and malignant human breast epithelial cell lines and correlation with ERα, PR and ERBB2 expression, and with invasiveness

To further assess the role of PKD1 in human breast cancer, we evaluated by qRT-PCR, as described in ‘Materials and methods’, the expression of *PRKD1* gene (encoding PKD1) in 7 non-cancerous breast epithelial cell lines and 31 breast cancer cell lines. PKD1 mRNA expression was absent (Ct > 35) in all non-cancerous breast cell lines (184B5, HMEC, hTert HME1, MCF-10A, MCF-10-2A, MCF-12A and MCF-12F; Table 1). On the other hand, 25.8% (8/31) of the breast cancer cell lines (BT474, HCC-202, Hs 578T, MCF-7, MDA-MB-134 VI, MDA-MB-415, MDA-MB-436 and PMC42) expressed marked PKD1 mRNA levels (Table 1).

**Table 1:** Analysis of PKD1 expression in human non-cancerous breast epithelial cells and breast cancer cell lines by qRT-PCR

Cell line	PKD1 expression (mRNA level)
Non-cancerous breast epithelial cells	
184B5	Negative*
HMEC	Negative
hTert HME1	Negative
MCF-10A	Negative
MCF-10-2A	Negative
MCF-12A	Negative
MCF-12F	Negative
Breast cancer cells	
BT20	Negative
BT474	Positive (45)†
BT483	Negative
BT549	Negative
CAMA1	Negative
HBL100	Negative
HCC-1143	Negative
HCC-1187	Negative
HCC-1428	Negative
HCC-1500	Negative
HCC-1569	Negative
HCC-1599	Negative
HCC-1937	Negative
HCC-1954	Negative
HCC-202	Positive (47)
HCC-38	Negative
HCC-70	Negative
Hs 578T	Positive (165)
MCF-7	Positive (35)
MDA-MB-134 VI	Positive (49)
MDA-MB-157	Negative
MDA-MB-231	Negative
MDA-MB-361	Negative
MDA-MB-415	Positive (93)
MDA-MB-436	Positive (116)
MDA-MB-453	Negative
MDA-MB-468	Negative
PMC42	Positive (111)
SKBR3	Negative
T47D	Negative
ZR75-1	Negative

* Absence or very low levels of PKD1 mRNA, which were only detectable but not reliably quantifiable by means of real-time quantitative RT-PCR assays (Ct > 35). ^†^Marked levels of PKD1 mRNA (Ct < 35), that were reliably quantifiable with thus possibility of N_*PRKD1*_ value determination (between parenthesis). N_*PRKD1*_ values were next normalized such that the value for the “basal mRNA level” (smallest amount of target gene mRNA quantifiable, Ct = 35) was 1.

**Fig. 6:**
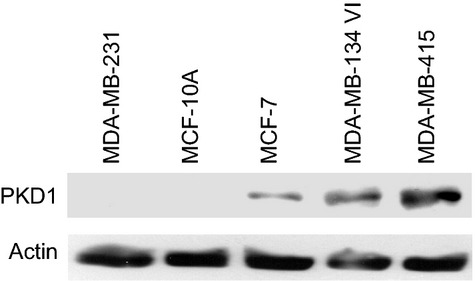
Analysis of PKD1 protein expression in breast cancer cell lines. Protein extracts from MDA-MB-231, MCF-10A, MCF-7, MDA-MB-134 VI and MDA-MB-415 were analysed by western blot by using anti-PKD1 or anti-actin antibodies.

Analysis of PKD1 protein expression in five of the cell lines tested by qRT-PCR showed a correlation between PKD1 mRNA and protein levels (Fig. 6). This result confirms the specificity of the primers used and suggests that PKD1 mRNAs are translated into functional PKD1 proteins.

Next, the relationship between PKD1 and ERα, PR, and ERBB2 statuses was explored. Four of the eight PKD1-positive breast cancer cell lines (50%) expressed high levels of ERα as compared to five of the 30 PKD1-negative breast cell lines (16.6%; Table 2). Thus, 76.3% (29/38) of the breast cell lines showed correlation regarding qualitative expression between PKD1 and ERα. However, no correlation was observed in these cell lines between PKD1 expression and PR expression or ERBB2 overexpression, neither quantitatively (Spearman correlation coefficient: *r* = 0.109; *P* = 0.52 (NS) and *r* = −0.169; *P* = 0.31 (NS) respectively) nor qualitatively (Table 2).

**Table 2:** Clinicopathological analysis of PKD1 positive and negative breast cancer cell lines

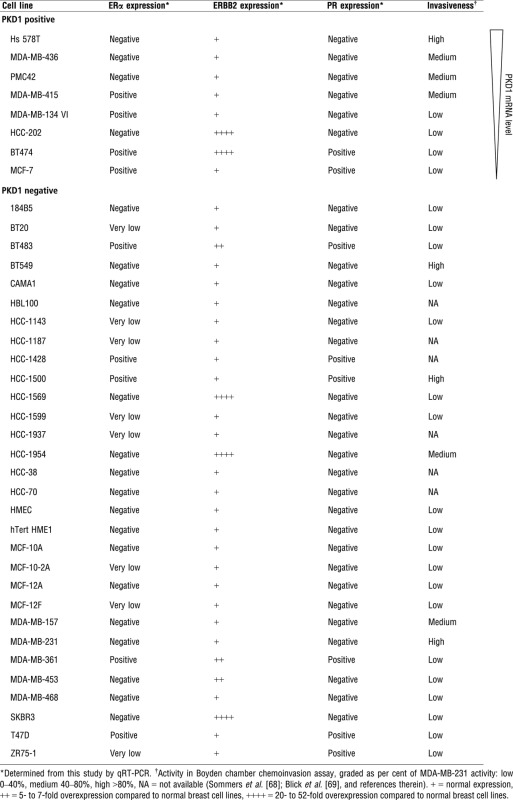

Interestingly, among the PKD1-positive cell lines tested (*n* = 8), a strong PKD1 expression was associated with a high invasive phenotype, whereas lower expression levels were found in weakly invasive breast cancer cells (Table 2). These results indicate a strong correlation between PKD1 expression and invasiveness in breast cancer cells (Mann–Whitney *U*-test *n* = 32 *P* < 0.0001) suggesting a role of PKD1 in breast cancer invasion.

### PKD1 knockdown reduces estrogen-independent and -dependent growth in MDA-MB-415 cell line

**Fig. 7:**
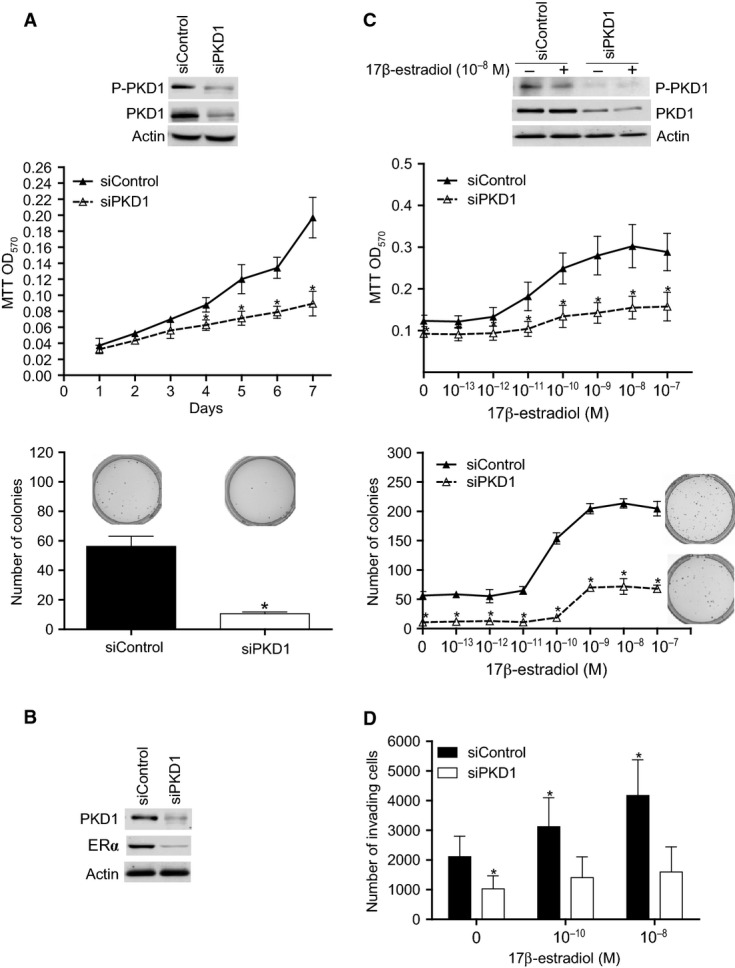
PKD1 knockdown reduces estrogen-dependent and -independent oncogenic properties of MDA-MB-415 cells. (**A**) MDA-MB-415 cells were transfected with siControl or siPKD1. The next day, transfected cells were seeded in estrogen-free medium for cell proliferation analysis by MTT assay (graph) or in estrogen-free methylcellulose for colony formation assay (photographs and histogram). The photographs presented are those of typical experiments. The results are the means ± SD for three independent experiments. *P < 0.01 *versus* siControl transfected cells. Inset: immunodetection of phospho-PKD1, PKD1 and actin after 4 days of transfection. (**B**) MDA-MB-415 cells transfected with siPKD1 or siControl were cultured for 3 days in estrogen-free medium and then analysed by western blot for PKD1, ERα and actin expression. The autoradiograms presented are those of a typical experiment. (**C**) Same experiments as in panel A except that the cells were cultured in the presence of different concentrations of 17β-oestradiol as indicated. The MTT assay was performed after 4 days of incubation and the photographs represent colonies formed in the presence of 10^−8^ M 17β-estradiol. The photographs and autoradiograms presented are those of typical experiments. The results are the means ± SD for three independent experiments. *P < 0.01 *versus* siControl transfected cells. (**D**) MDA-MB-415 cells were transfected with siControl or siPKD1. Three days after transfection, cells were incubated in phenol red-free medium containing 0, 10^−10^ or 10^−8^ M 17β-estradiol in the matrigel-coated upper well of a transwell chamber while in the lower well complete medium was added. After 48 hrs, cells that invaded to the lower well were counted. The results presented are the means ± SD for three independent experiments. *P < 0.01 *versus* siControl transfected cells cultured in absence of 17β-estradiol.

Now, we know the status of PKD1 in a wide range of breast cancer cell lines. To confirm the role of this kinase in ERα-positive breast cancer, we have chosen to test the effect of its down-regulation in MDA-MB-415 cell line which is ERα-positive, expresses high levels of PKD1 and is invasive (Table 2). First, to analyse the estrogen-independent role of PKD1, MDA-MB-415 cells were transfected with siControl or siPKD1 and cultured in estrogen-free medium for western blot, proliferation and anchorage-independent growth assays. As shown in Figure 7A (inset), PKD1 is expressed and phosphorylated (active) in control cells and depleted in siPKD1 transfected cells. In contrast to control MCF-7 cells (Figs 1 and 5), MDA-MB-415 cells can grow dependently (Fig. 7A, graph) and independently (Fig. 7A, histogram) of anchorage in estrogen-free medium. Interestingly, MDA-MB-415 proliferation and anchorage-independent growth were strongly decreased by PKD1 knockdown (siPKD1; ∼54.8% and 81.3% reduction in cell and colony number respectively). Therefore, these data suggest that PKD1 increases estrogen-independent growth of MDA-MB-415 breast cancer cells.

As PKD1 increases ERα levels in MCF-7 cells (Fig. 3), we also tested whether PKD1 knockdown affects ERα expression in MDA-MB-415 cells. As shown in Figure 7B, siPKD1 transfected cells expressed less ERα levels than siControl transfected ones. Moreover, proliferation and anchorage-independent growth were dose-dependently enhanced by 17β-estradiol treatment (Fig. 7C). PKD1 knockdown significantly reduced 17β-estradiol-induced proliferation (∼49.1% decrease in cell number after 7 days of culture with 10^−8^ M 17β-estradiol) and anchorage-independent growth (∼66.4% decrease in colony number at 10^−8^ M; Fig. 7C). These data indicate that PKD1 enhances 17β-estradiol-induced proliferation in MDA-MB-415 breast cancer cells.

### PKD1 knockdown reduces estrogen-dependent and -independent invasion in MDA-MB-415 cell line

As a strong correlation between PKD1 expression and invasiveness was observed in PKD1-positive breast cancer cells (Table 2), the role of PKD1 in the invasiveness of ERα-positive breast cancer cells was analysed. By using the transwell system, we show that ∼2200 MDA-MB-415 cells cultured in the upper chamber in phenol red-, FBS- and 17β-estradiol-free DMEM medium invaded through the matrigel to the lower chamber containing complete medium (Fig. 7D). PKD1 knockdown decreased by ∼51.46% the invasion of MDA-MB-415 cells when no 17β-estradiol was added to the upper chamber (Fig. 7D). Addition of 10^−10^ or 10^−8^ M 17β-estradiol to the upper chamber increased the invasion of siControl transfected cells by 1.5 and 2-fold respectively but did not significantly affect the invasion of siPKD1 transfected cells (Fig. 7D) in which PKD1 and ERα levels were strongly decreased (Fig. 7B). Taken together, these results suggest that PKD1 knockdown inhibits estrogen-independent and -dependent invasion of MDA-MB-415 cells.

By contrast, in non-invasive MCF-7 cells, neither PKD1-overexpressing (P cells) nor PKD1-depleted (C cells transfected with siPKD1) cells did invade (data not shown).

### PKD1 mRNA expression in human ERα-positive breast tumours and relationship with tamoxifen responsiveness and classical clinicopathologic prognostic factors

**Fig. 8:**
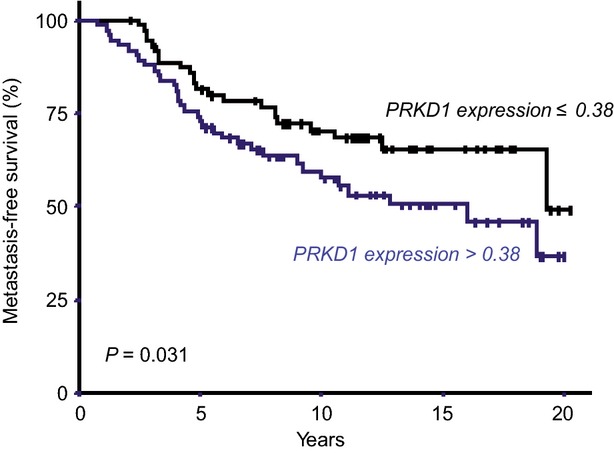
High PKD1 mRNA levels are associated with poor prognosis. Metastasis-free survival (MFS) curves for tamoxifen-treated patients with high (N_*PRKD1*_ > 0.38) and low (N_*PRKD1*_ ≤ 0.38) tumoral expression levels of PKD1.

Since PKD1 overexpression confers resistance to antioestrogen ICI 182,780 in MCF-7 ERα-positive breast cancer cells (Fig. 4), we investigated the possible relationship between PKD1 expression level and patient responsiveness to the most common antiestrogen agent in clinical use, *i.e*. tamoxifen. Therefore, PKD1 mRNA levels were quantified by qRT-PCR in 152 primary ERα-positive breast tumours from patients treated with primary surgery followed by adjuvant tamoxifen alone. MFS distributions were estimated by the Kaplan-Meier method. Area under the curve analyses were performed to identify a cut-point which divides the cohort into relevant *PRKD1* expression subgroups. Consequently, when compared with tumours expressing low PKD1 mRNA levels ≤0.38 (*n* = 75, 49.34%), tumours with high PKD1 mRNA expression >0.38 (*n* = 77, 50.66%) were significantly associated with less MFS (*P* = 0.031; Fig. 8). In fact, patients with poor prognosis overexpressed *PRKD1* [7.5-year MFS 65.5% (59.6–75); 15-year MFS 52.1% (39–63.4)], while those with good prognosis had lower *PRKD1* expression [7.5-year MFS 77.9% (67–85.9); 15-year MFS 68.8% (56.5–78.9)].

To determine whether the prognostic value of PKD1 was dependent on classical clinicopathological prognostic factors, the possible relationship between high PKD1 mRNA expression (>0.38) and clinicopathologic variables was analysed. Except for lymph node status (28.6% of lymph node-negative tumours express high levels of PKD1 mRNA as compared to 55.6% of the lymph node-positive tumours, *P* = 0.034), no relationship was found between the expression pattern of PKD1 mRNA and most classical pathological and clinical parameters (age, SBR histological grade, macroscopic tumour size, PR and ERBB2 statuses and PIK3CA mutation status; Table 3). Therefore, taken all together, these results suggest that PKD1 may represent a novel attractive prognostic factor for hormone responsiveness and metastasis in ERα-positive breast cancer.

**Table 3:** High PKD1 mRNA expression (>0.38 relative to normal breast tissues) and relation to standard clinicopathological factors

		Number of patients (%)		
	Total population (%)	PKD1 mRNA expression ≤0.38	PKD1 mRNA expression >0.38	P*
Total	152 (100.0)	75 (49.3)	77 (50.7)	
Age				
≤60	18 (11.8)	10 (13.3)	8 (10.4)	0.57 (NS)
>60	134 (88.2)	65 (86.7)	69 (89.6)	
SBR histological grade†				
I	25 (16.4)	8 (10.7)	17 (22.1)	0.081 (NS)
II	93 (61.2)	52 (69.3)	41 (53.2)	
III	34 (22.4)	15 (20)	19 (24.7)	
Lymph node status				
0	28 (18.4)	20 (26.7)	8 (10.4)	**0.034**
1–3	96 (63.2)	43 (57.3)	53 (68.8)	
>3	28 (18.4)	12 (16)	16 (20.8)	
Macroscopic tumour size‡				
≤25 mm	81 (54.4)	38 (51.4)	43 (57.3)	0.46 (NS)
>25 mm	68 (45.6)	36 (48.6)	32 (42.7)	
PR status				
Negative	45 (29.6)	27 (36)	18 (23.4)	0.088 (NS)
Positive	107 (70.4)	48 (64)	59 (76.6)	
ERBB2 status				
Negative	130 (85.5)	64 (85.3)	66 (85.7)	0.95 (NS)
Positive	22 (14.5)	11 (14.7)	11 (14.3)	
PIK3CA mutation status				
Wild-type	89 (58.6)	49 (65.3)	40 (51.9)	0.094 (NS)
Mutated	63 (41.4)	26 (34.7)	37 (48.1)	

* Chi-squared test,

† Scarff Bloom Richardson classification,

‡ information available for 149 patients.

### PKD1 mRNA expression positively correlates with vimentin and EGFR mRNA expression in breast tumours

Since PKD1 expression was correlated with the invasive phenotype of breast cancer cell lines (Table 2) and since its highest expression level was observed in a mesenchymal-like breast cancer cell line (*i.e*. Hs578T), the relationship between PKD1 expression levels and EGFR, vimentin and E-cadherin levels was analysed. Spearman correlation coefficient analysis indicates that PKD1 mRNA levels are strongly linked to EGFR and vimentin levels (*r* = 0.622; *P* < 0.0000001 and *r* = 0.767; *P* < 0.0000001, respectively) but not to E-cadherin levels (*r* = −0.034; *P* = 0.68 (NS); Table 4). These data further strengthen a crucial role of PKD1 in invasion.

**Table 4:** Relationship between PKD1 mRNA levels and EGFR, vimentin and E-cadherin levels in the 152 breast tumours

	PKD1
EGFR	
Spearman correlation coefficient	0.622
*P*	<0.0000001
Vimentin	
Spearman correlation coefficient	0.767
*P*	<0.0000001
E-cadherin	
Spearman correlation coefficient	−0.034
*P*	0.68 (NS)

## Discussion

We have previously reported that protein kinase D1 stimulates proliferation and oncogenic properties of estrogen-dependent MCF-7 breast cancer cells [6]. This prompted us to search, in the present study, whether this serine/threonine kinase operates in this cell line through an estrogen/ERα-dependent or -independent pathway. PKD1 overexpression decreased by 10- and 100-fold the concentrations of 17β-estradiol required for MCF-7 cell proliferation and anchorage-independent growth, respectively, and exerts with 17β-estradiol a synergistic stimulatory effect. Since this higher sensitivity to 17β-estradiol could reflect modulation of ERα content [27,28], the expression pattern and the phosphorylation state of ERα were analysed in PKD1-overexpressing cells and were found markedly increased suggesting that the newly synthesized receptors are fully activated and may therefore exert their pleiotropic roles. Such increase in ERα expression could be the result of a PKD1-stimulated transcriptional activity of the *ESR1* gene (encoding ERα). In fact, PKD1 regulates gene transcription, either by phosphorylation and activation of transcription factors [29] or by phosphorylation and induction of the nuclear export of histone deacetylases (HDACs) [30–32]. Since HDAC inhibition induces the expression of ERα in ERα-negative mammary tumour cells [33], one may hypothesize that HDACs could be involved in the regulation of ERα expression. Nevertheless, we cannot exclude that PKD1 could also modulate either the activity of other *ESR1*-inducing transcription factors such as Sp1 [34] and GATA-3 [35] or ERα degradation by the ubiquitin-proteasome pathway [36]. Such hypotheses are currently under investigation.

Interestingly, whatever overexpressed or not, endogenous PKD1 also plays a major role in ERα expression since PKD1 depletion strongly inhibited ERα expression in control MCF-7 and MDA-MB-415 cells. Such important role of PKD1 was further supported by Eiseler *et al*. [37] who showed, in agreement with our present data, that PKD1 is expressed in ERα-positive (BT-474 and MCF-7) but absent in ERα-negative (SKBR3 and MDA-MB-231) breast cancer cell lines. By a large-scale analysis of PKD1 and ERα mRNA expression realized in 38 breast cell lines, we further strengthen the notion that a strong qualitative correlation (76.3%) exists between PKD1 and ERα. However, 10.5% of the cell lines tested were PKD1-positive/ERα-negative and 13.1% were PKD1-negative/ERα-positive. Furthermore, PKD1 expression in PKD1-negative/ERα-negative MDA-MB-231 cells did not induce ERα expression (data not shown). These suggest, on the one hand that PKD1 is not always sufficient per se to allow ERα expression in all cell lines and on the other hand that ERα can also be expressed independently of PKD1. However, independently of the precise relationship that exists between these two partners and that needs to be characterized, our results put in evidence a strong link between PKD1 and ERα expression.

For the first time to our knowledge, we also demonstrated that PKD1 overexpression allows MCF-7 cells to proliferate and survive dependently and independently of anchorage, in an estrogen- and ERα-independent manner. In fact, although PKD1-overexpressing cells are sensitive to ICI 182,780 because of their increased sensitivity to 17β-estradiol, these cells, in contrast to control cells, are still able to significantly proliferate and survive in presence of this antiestrogen and/or in estrogen-free conditions. Interestingly, we observed that ERα-positive MDA-MB-415 breast cancer cells, that express high endogenous PKD1 levels, are also able to proliferate and survive in estrogen-free medium. By siPKD1 transfection, we confirmed that PKD1 may be responsible, at least in part, for this estrogen-independent growth. This newly described property of PKD1 to confer independence to estrogens is of major importance since independence to these hormones characterizes more aggressive breast cancers which are unresponsive to antiestrogen therapy [38]. Consistent with these *in vitro* data, our *in vivo* analysis of PKD1 expression in 152 ERα-positive breast tumours from tamoxifen-treated patients showed that high PKD1 expression is associated with less tamoxifen responsiveness. Therefore, PKD1-overexpressing cells seem to have acquired new pro-proliferative and survival signalling pathways [6]. Since ICI 182,780 suppresses ERα expression, this PKD1-induced independence to estrogens is not the consequence of a ligand-independent activation of ERα [39–44]. However, one may suppose that PKD1 overexpression may induce ERα targeted signalling pathways such as receptor tyrosine kinase (RTK)/PI 3-kinase/Akt or RTK/MEK/ERK [45–47]. This last pathway seems to be the most relevant for PKD1-induced resistance to antiestrogens since PKD1 activates ERK1/2 in MCF-7 cells [6,48]. Consistently, clinical data showed that ERK1/2 expression and/or activity correlates with absence of response to antiestrogens and reduction in patient survival [49,50]. Moreover, overexpression of EGFR and HER2, whose ligand (*i.e*. the epidermal growth factor) is capable of inducing PKD1 activity [51], was observed in antiestrogen-resistant breast cancers [52,53].

Our present wide prospective analysis of PKD1 expression pattern in normal and malignant breast tissues and cell lines also shows that PKD1 expression strongly correlates with invasion and epithelial-mesenchymal transition markers (i.e. EGFR and vimentin) commonly associated with increased aggressiveness and invasive and metastatic potential [54]. Furthermore, the highest expression levels of PKD1 were not only observed in Hs 578T, a mesenchymal-like and highly invasive breast cancer cell line, but were also associated in breast tumours with poor prognosis (less MFS) identifying PKD1 as a potential marker of aggressive and highly invasive breast cancer. This may appear discrepant with a previous study that identified decreased PKD1 expression as a marker for invasive breast cancer [37]. However, this study was performed on tissue microarray (TMA) slides including 10 normal breast tissues and only 50 invasive carcinoma samples and 6 breast cell lines (SKBR3, T47D, MDA-MB-231, BT474, MCF-7 and MCF-10A) among which only one, i.e. MDA-MB-231, is invasive, whereas the others are lowly invasive [37]. Furthermore, most PKD1 antibodies are described not to be totally specific which can interfere with the proper PKD1 labelling in TMA assays. Therefore, based on our current large-scale analysis, realized in 152 malignant breast tissues and in 38 non-cancerous and cancerous breast cell lines, and by using a more sensitive and specific method (qRT-PCR) [55], we currently present PKD1 as a new pertinent marker for breast cancer invasion. To confirm this hypothesis, we analysed the putative invasive role of PKD1 in ERα-positive MCF-7 and MDA-MB-415 cell lines. In non-invasive and low PKD1-expressing MCF-7 cells, neither PKD1 overexpression nor PKD1 knockdown affected cell invasion, suggesting that parameters crucial for invasion are absent in this cell line and cannot be replaced by PKD1 overexpression. In fact, pro-invasive markers such as vimentin, MMP2 and MMP3 were hardly detectable in MCF-7 cells, whereas E-cadherin (an anti-invasive marker) was strongly expressed (data not shown). Furthermore, PKD1 overexpression in MCF-7 cells did not affect the expression of any of these proteins crucial for invasion regulation (data not shown). However, in invasive and high PKD1-expressing MDA-MB-415 cells, PKD1 knockdown strongly inhibited 17β-estradiol-induced and -independent invasion which further confirms the estrogen-dependent and -independent role of PKD1 in breast cancer progression. Many other data support the invasive role of PKD1. In fact, the invasiveness of breast cancer cells correlates with the formation of a ternary complex between PKD1, cortactin and paxillin in invadopodia-enriched membranes [56]. Furthermore, PKD1 has been found to exert pro-migratory and -invasive role in many cell types such as intestinal epithelial cells [57], fibroblasts [58] and pancreatic cancer cells [59], and to be implicated in several pathways mediating cell migration and invasion. For example, PKD1 regulates fibroblast motility through modulation of anterograde membrane traffic from the trans-Golgi network to the plasma membrane [60], affects cell migration by transporting αvβ3 integrin to focal adhesions [58], and regulates the expression or activity of several proteins important for cell migration and invasion such as cortactin [61], Snail1 [62], Slingshot 1 like [63] and MMPs [37,64]. However, a recent study by Borges and colleagues showed that re-expression of PKD1 reduces the invasive potential of the PKD1-negative MDA-MB-231 breast cancer cells [65]. Since this cell line is ERα-negative, the results from their study and our present study suggest that PKD1 may have different roles in the regulation of breast cancer invasion depending on the expression or not of ERα. Therefore, the molecular subtype of breast cancer should be determined and taken into account before any prognosis and/or treatment decision.

Finally, we showed that PKD1 expression does not correlate with classical clinical and pathological prognostic factors (SBR histological grade, macroscopic tumour size, PR and ERBB2 statuses and PIK3CA mutation status). This interesting point suggests that PKD1 may also represent a novel attractive independent prognostic factor in breast cancer as recently described for esophageal squamous [66] and laryngeal [67] cancers.

In conclusion, our study indicates that PKD1 expression might be critical, on the one hand, to the initiation of ERα-positive breast cancers and, on the other hand, to the progression to a more advanced tumour. Therefore, we suggest that PKD1 may become a new potential therapeutic target for initiated and advanced breast cancer treatment that may counteract endocrine therapy-resistance.
